# Assessment of Community Awareness and Practices Concerning Indoor Air Pollutants — Madison County, Alabama, June 2017

**DOI:** 10.15585/mmwr.mm6715a3

**Published:** 2018-04-20

**Authors:** Charlene Siza, Melissa Morrison, Scott Harris, Timothy Hatch, Michael Tyler

**Affiliations:** ^1^Epidemic Intelligence Service, CDC; ^2^Bureau of Communicable Disease, Alabama Department of Public Health; ^3^Division of State and Local Readiness, Office of Public Health Preparedness and Response, CDC; ^4^Center for Emergency Preparedness, Alabama Department of Public Health; ^5^Office of the State Health Officer, Alabama Department of Public Health.

The Alabama Department of Public Health (ADPH) conducts an annual community assessment to evaluate household preparedness and local public health concerns. In June 2017, ADPH conducted a Community Assessment for Public Health Emergency Response (CASPER), focusing on indoor air pollutants in seven neighborhoods in Madison County, Alabama, where a large percentage of homes were built before 1980. Local health partners had concerns about indoor air quality and environmental risks such as radon; however, limited information was available regarding community awareness, prevention, and mitigation measures related to potential exposures. Weighted response frequencies were calculated from assessment responses. Among 192 household interview respondents, 78.4% were aware of potential indoor lead exposures, but only 12.6% of respondents living in houses built before 1978 reported that the house had been tested for lead. Similarly, respondents in 70.2% of households had heard of radon; however, only 7.3% of houses had been tested for radon. Smoking was reported by residents of 45.7% of households; among those, 48.4% reported that smoking occurred inside the house. Identified gaps in exposure prevention and mitigation, including low lead and radon testing rates and a high prevalence of indoor smoking, were shared with the local health department, and recommendations for timely interventions and policy guidance (e.g., targeted education campaigns and smoking cessation programs) were presented. Results of this CASPER demonstrated its usefulness and efficiency in gathering community-level data to help guide public health policies and timely interventions.

According to ADPH’s Radon Program, Madison County’s underground geology, which allows radon gas to accumulate and more readily enter houses and other buildings above ground, places it at high risk for elevated radon levels (*1*,*2*). The sampling frame for the CASPER included seven neighborhoods identified by community partners as having a majority of homes built before 1980. Census blocks that included these neighborhoods were obtained from 2010 U.S. Census data, which indicated that the sampling frame included 1,772 occupied houses with 4,486 residents. CASPER methodology was used (*3*); 30 census blocks were selected randomly from a total of 78 blocks, with the probability of selection proportional to the number of housing units in that block (hereafter referred to as clusters). Within each cluster, seven households were selected for interviews using systematic random sampling, for a target of 210 interviews. If one of the original seven households was not available or the residents refused to participate, systematic random sampling was used to select another household. Two-person interview teams conducted interviews with one respondent aged ≥18 years from each selected household. Contact was attempted at 407 households, and successful contact with a respondent was made at 281. Overall, 192 (91.4%) of the targeted 210 surveys were completed, representing a response rate* of 47.2% and a cooperation rate^†^ of 68.3%.

The questionnaire for this assessment was adapted from previous CASPERs and established surveillance systems, including the Behavioral Risk Factor Surveillance System. Because of the reported high risk for exposure to indoor air pollutants, including lead and radon, in the selected neighborhoods, a special topics section was added to the questionnaire that focused on knowledge and prevention practices related to indoor air pollutants and included questions on exposure to tobacco smoke, mold, dust, and relevant respiratory health conditions in any household member.

Data were analyzed to obtain response frequencies for each question. Analysis was weighted to account for the complex sampling method and more accurately represent the sampling figure (*3*). Weighted percentages are reported.

Awareness of potential lead exposures in older homes was reported by residents of 78.4% of households (Table 1). Among 86 houses built before 1978, residents in 12.6% reported that lead testing had been conducted. Although no positive test results were reported, respondents in 29.3% of these households did not know or refused to report whether their homes had been tested. Overall, 14 (6.7%) respondents reported that a household resident had been previously tested for increased blood lead levels; among those tested, two were reported to have an elevated (≥5 *μ*g/dL) blood lead level (weighted percentage 14.3%) (Table 1).

**TABLE 1:** Awareness of potential for household lead exposure among survey respondents to a Community Assessment for Public Health Emergency Response, Madison County, Alabama, June 2017

Characteristic	No. of households (%)	Estimated no. of households*	Weighted % (95% CI)
**Respondent aware of possible lead sources in older homes (n = 192)**			
Yes	152 (79.2)	1,389	78.4 (76.4–80.2)
No	31 (16.1)	286	16.1 (14.5–17.9)
Don’t know or refused	9 (4.7)	97	5.5 (4.5–6.6)
**Was house tested for lead?^†^**			
Yes	11 (12.8)	99	12.6 (10.4–15.1)
No	52 (60.5)	458	58.1 (54.6–61.5)
Don’t know or refused	23 (26.7)	231	29.3 (26.3–32.6)
**House tested positive for lead (n = 11)**			
Yes	0	—	—
No	8 (72.7)	74	74.5 (65.0–82.9)
Don’t know or refused	3 (27.3)	25	25.5 (17.1–35.0)
**Household resident tested for elevated blood lead levels**			
Yes	14 (7.3)	118	6.7 (5.6–7.9)
No	161 (83.9)	1486	83.9 (82.1–85.5)
Don’t know or refused	17 (8.8)	168	9.5 (8.2–10.9)
**Elevated blood lead level**^§^ **in tested household resident (n = 14)**			
Yes	2 (14.3)	17	14.3 (8.6–22.0)
No	9 (64.3)	76	64.3 (55.1–73.0)
Don’t know or refused	3 (21.4)	25	21.4 (14.2–29.7)

**Abbreviation:** CI = confidence interval.

* Obtained by weighting the frequencies. The weight for each cluster was calculated by dividing the total number of housing units in the sampling frame by the product of the number of housing units interviewed within the cluster and the number of clusters selected.

^†^ Only asked for houses built before 1978 (N = 86).

^§^ ≥5 *μ*g/dL.

Respondents in 70.2% of households reported awareness of radon (Table 2). Although 87.8% of household respondents who reported awareness of radon agreed with the statement that prolonged exposure to radon could be harmful, only 23.9% were aware that prolonged radon exposure could cause lung cancer (*4*), and only 17.1% of household respondents were aware that a free radon test kit could be requested from the health department. Among 131 respondents reporting awareness of radon, 7.3% stated that their homes had already been tested.

**TABLE 2:** Awareness of potential for household radon exposure among survey respondents to a Community Assessment for Public Health Emergency Response, Madison County, Alabama, June 2017

Characteristic	No. of households (%)	Estimated no. of households*	Weighted % (95% CI)
**Respondent had heard of radon (n = 192)**			
Yes	131 (68.2)	1,244	70.2 (68.0–72.3)
No	59 (30.7)	511	28.8 (26.8–31.0)
Don’t know or refused	2 (1.0)	17	1.0 (0.6–1.5)
**Respondent agreed that prolonged radon exposure can be harmful (n = 131)**			
Agree	113 (86.3)	1092	87.8 (85.9–89.5)
Disagree	0	—	—
Neither agree nor disagree	6 (4.6)	51	4.1 (3.1–5.3)
Don’t know or refused	12 (9.2)	101	8.1 (6.7–9.8)
**Respondent beliefs about possible health effects of radon (n = 113)**			
Lung cancer	28 (24.8)	261	23.9 (21.4–26.5)
Respiratory concerns	9 (8.0)	97	8.9 (7.3–10.7)
Other	12 (10.6)	120	11.0 (9.3–13.0)
Don’t know or no answer	64 (56.6)	614	56.2 (53.3–59.2)
**Respondent aware that ADPH offers free radon test kit (n = 131)**			
Yes	19 (14.5)	212	17.1 (15.1–19.3)
No	105 (80.2)	954	76.7 (74.2–78.9)
Don’t know or refused	7 (5.3)	78	6.3 (5.1–7.8)
**Was house tested for radon? (n = 131)**			
Yes	10 (7.6)	91	7.3 (6.0–8.9)
No	95 (72.5)	921	74.1 (71.6–76.4)
Don’t know or refused	26 (19.9)	232	18.7 (16.6–20.9)
**House had elevated radon levels (n = 10)**			
Yes	1 (10)	8	9.3 (3.9–16.7)
No	6 (60.0)	57	62.8 (51.9–72.6)
Don’t know or refused	3 (30.0)	25	27.9 (18.8–38.1)
**Plans to test house for radon in the next year (n = 131)**			
Yes	13 (9.9)	113	9.1 (7.6–10.8)
No	66 (50.4)	631	50.7 (48.0–53.5)
Don’t know or refused	52 (39.7)	500	40.2 (37.5–42.9)

**Abbreviations:** ADPH = Alabama Department of Public Health; CI = confidence interval.

* Obtained by weighting the frequencies. The weight for each cluster was calculated by dividing the total number of housing units in the sampling frame by the product of the number of housing units interviewed within the cluster and the number of clusters selected.

Among other self-reported indoor pollutant exposures, excessive dust was most commonly reported (22.4% of households) (Table 3). Respondents in 45.7% of households reported any current smoking by at least one household member, and among these, 48.4% reported that smoking occurred indoors (22.1% of all households).

**TABLE 3:** Selected self-reported indoor pollutant exposures, smoking status, health conditions, and symptoms **—** Community Assessment for Public Health Emergency Response, Madison County, Alabama, June 2017

Characteristic*	No. of households (%)	Estimated no. of households^†^	Weighted % (95% CI)
**Selected indoor pollutant exposures (n = 192)** ^§^			
Excessive dust	42 (21.9)	397	22.4 (20.5–24.4)
Excessive moisture	18 (9.4)	183	10.3 (9.0–11.8)
Mold growth	17 (8.9)	181	10.2 (8.9–11.7)
Unusual odors	16 (8.3)	148	8.3 (7.1–9.7)
None of the above	117 (60.9)	1,068	60.3 (58.0–62.5)
Don’t know or refused	18 (9.4)	158	8.9 (7.7–10.4)
**Household has a current smoker (n = 192)**			
Yes	84 (43.8)	811	45.7 (43.4–48.1)
No	106 (55.2)	945	53.3 (51.0–55.6)
Don’t know or refused	2 (1.0)	17	1.0 (0.6–1.5)
**Smoker smokes inside the house (n = 84)**			
Yes	41 (48.8)	392	48.4 (45.0–51.9)
No	43 (51.2)	418	51.6 (48.2–55.0)
**Health care provider diagnosed one of these conditions in household member (n = 192)**			
Allergies	84 (43.8)	798	45.0 (42.7–47.4)
Asthma	44 (22.9)	381	21.5 (19.7–23.5)
COPD	11 (5.7)	99	5.6 (4.6–6.8)
Emphysema	8 (4.2)	71	4.0 (3.2–5.0)
Lung cancer	3 (1.6)	46	2.6 (2.0–3.5)
None of the above	85 (44.3)	774	43.7 (41.4–46.0)
Don’t know or refused	3 (1.6)	25	1.4 (1.0–2.1)
**Household member experienced these conditions/symptoms in past 12 months (n = 192)**			
Allergies	114 (59.4)	1,030	58.1 (55.8–60.4)
Migraine	48 (25.0)	449	25.3 (23.3–27.4)
Sinus infection	42 (21.9)	403	22.7 (20.9–24.8)
Sore throat	38 (19.8)	379	21.4 (19.5–23.4)
Wheezing or asthma attack	38 (19.8)	337	19.0 (17.2–20.9)
Conjunctivitis	33 (17.2)	322	18.2 (16.4–20.0)
Bronchitis	29 (15.1)	267	15.1 (13.5–16.8)
Laryngitis	11 (5.7)	99	5.6 (4.6–6.8)
None of the above	42 (21.9)	395	22.3 (20.4–24.3)
Don’t know or refused	5 (2.6)	42	2.4 (1.8–3.2)

**Abbreviations:** CI = confidence interval; COPD = chronic obstructive pulmonary disease.

* Characteristics based on self-report.

^†^ Obtained by weighting the frequencies. The weight for each cluster was calculated by dividing the total number of housing units in the sampling frame by the product of the number of housing units interviewed within the cluster and the number of clusters selected.

^§^ Excessive dust, excessive moisture, and unusual odors based on respondent’s subjective report. Mold growth was defined as larger than the size of a $1 bill.

The most frequently reported respiratory diagnoses among respondents were allergies (45.0%) and asthma (21.5%). Diagnosed chronic obstructive pulmonary disease (COPD) (5.6%), emphysema (4.0%), and lung cancer (2.6%) were also reported. These conditions were reported separately as seen in other respiratory surveys, despite clinical and pathologic overlap. In the preceding 12 months, 58.1% of household respondents reported a resident who experienced allergies (Table 3).

## Discussion

Among Madison County households at risk, interviews identified gaps in respondent knowledge and protective behaviors related to indoor air pollutants. A majority of household respondents reported being aware that lead exposure could exist in older homes; however, respondents in only a small percentage of houses built before 1978, when lead-based paint was banned for residential use, reported that their homes had been tested. Lead-based hazards from paint chips or an accumulation in dust or soil are more common in older homes (*5*). Creation of lead dust by sanding surfaces or removing old paint presents a hazard during home remodeling or renovation (*6*). This might not only pose a risk to household residents, but also an occupational risk to workers who perform these renovations.

A second important indoor air pollutant is radon, a naturally occurring radioactive gas that is the second leading cause of lung cancer after cigarette smoking (*4*). Madison County is an area with a high potential for elevated radon levels (*2*). The majority of household respondents reported awareness of radon, but fewer than a quarter knew that it could cause lung cancer. More importantly, few houses had been tested for elevated indoor radon levels. Although free test kits are available through the health department, few respondents knew of this service.

In addition to possible harmful exposures related to lead and radon in the home, respondents in nearly half of households reported that at least one resident in the home smoked, and in almost half of these houses, smoking indoors was reported. Smoking, especially indoors, might result in exposure of other household members to secondhand smoke, which is known to increase the risk for cancer, respiratory diseases, and cardiovascular diseases including stroke (*7*). Educating residents about the dangers of secondhand smoke exposure and the benefits of implementing smoke-free rules in their households is an important intervention that, along with smoking cessation and support programs, can improve health in these neighborhoods (*8*).

Interviews with members of households indicated that, among respiratory conditions diagnosed by health care providers, allergies and asthma were the most prevalent although other severe conditions such as emphysema, COPD, and lung cancer also were reported. Although causality cannot be inferred from this analysis, information on respiratory conditions prevalent in these areas could be used to help prioritize interventions potentially related to indoor air pollutant exposures.

The findings of this report are subject to at least two limitations. First, although households were systematically selected, participation was voluntary, and the findings might be subject to response and social desirability biases, which could overestimate the prevalence of certain health conditions or reported knowledge about indoor pollutants. Second, the information gathered by the CASPER is only representative of the sampling frame chosen and cannot be used to draw conclusions about other communities or regions in Alabama.

Conducting a community assessment is a relatively rapid and inexpensive way to obtain a better understanding of the current health-related needs of a community. This assessment obtained data on knowledge and prevention practices related to indoor air pollutants in neighborhoods known to be at increased risk and suggested that community health interventions to raise awareness of the importance of testing homes for lead and to provide educational resources to reduce lead exposure risks when remodeling older homes are needed. In addition, the findings provide evidence that public health programs need to increase awareness of radon testing and mitigation options in these neighborhoods with a high risk for radon exposure as well as provide smoking cessation options and education about secondhand smoke effects. Community-specific data can aid policy makers and local or federal partners in developing and implementing targeted interventions.

**Summary**

**What is already known about this topic?**

Community Assessment for Public Health Emergency Response (CASPER) is a household-level rapid assessment commonly used during disasters and emergency preparedness planning.

**What is added by this report?**

The CASPER conducted among 192 households in Madison County, Alabama, about selected indoor air pollutants and routine emergency preparedness found the majority of residents were aware of potential indoor lead exposures and had heard of radon, but most had not tested for either. Smoking inside the house occurred among 22% of households.

**What are the implications for public health practice?**

Using the CASPER methodology in nondisaster settings to collect community-specific data can guide targeted intervention and prevention recommendations for local public health departments and their community partners.

## Footnotes

* Response rate is calculated as the number of completed interviews divided by the number of households where contact was attempted.

† Cooperation rate is calculated as the number of completed interviews divided by the number of households where contact with a respondent was made.
